# Mixed Model Association Mapping for Fusarium Head Blight Resistance in Tunisian-Derived Durum Wheat Populations

**DOI:** 10.1534/g3.111.000489

**Published:** 2011-08-01

**Authors:** Farhad Ghavami, Elias M. Elias, Sujan Mamidi, Omid Ansari, Mehdi Sargolzaei, Tika Adhikari, Mohamed Mergoum, Shahryar F. Kianian

**Affiliations:** * Department of Plant Sciences and; † Department of Plant Pathology, North Dakota State University, Fargo, North Dakota 58108; ‡ Center for Genetic Improvement of Livestock, University of Guelph, Guelph, Ontario N1G 2W1, Canada; § Grains Research and Development Corporation (GRDC), Barton, Australian Capital Territory 2600, Australia

**Keywords:** association mapping, durum wheat, Fusarium head blight, QTL analysis, suppressor of resistance

## Abstract

Sources of resistance to Fusarium head blight (FHB) in wheat are mostly restricted to Chinese hexaploid genotypes. The effort to incorporate the resistance from hexaploid wheat or wild relatives to cultivated durum wheat (*Triticum turgidum* L. var. *durum* Desf.) have not been successful in providing resistance to the level of the donor parents. In this study, we used 171 BC_1_F_6_ and 169 BC_1_F_7_ lines derived from crossing of four Tunisian tetraploid sources of resistance (Tun7, Tun18, Tun34, Tun36) with durum cultivars ‘Ben,’ ‘Maier,’ ‘Lebsock,’ and ‘Mountrail’ for association studies. The Tun18 and Tun7 FHB resistances were found to be comparable to the best hexaploid wheat sources. A new significant QTL for FHB resistance was identified on the long arm of chromosome 5B (*Qfhs.ndsu-5BL*) with both association and classical QTL mapping analysis. Linkage disequilibrium (LD) blocks extending up to 40 cM were evident in these populations. The linear mixed model considering the structure (Q or P) and the kinship matrix (K_T_) estimated by restricted maximum likelihood (REML) was identified as the best for association studies in a mixture of wheat populations from a breeding program. The results of association mapping analysis also demonstrated a region on the short arm of chromosome 3B as potentially linked to FHB resistance. This region is in proximity of major FHB resistance gene *fhb1* reported in hexaploid wheat. A possibility of having susceptibility or suppressor of resistance gene(s) on durum wheat chromosome 2A was further confirmed in this material, explaining the problem in developing resistant genotypes without counter selection against this region.

*Fusarium* head blight (FHB), caused mainly by *Fusarium graminearum* Schwabe, is one of the most devastating diseases of wheat (*Triticum aestivum* L.) worldwide (Cuthbert *et al.* 2007; Liu and Anderson 2003). Host resistance is considered as the most effective method to control FHB. But the efforts in this area have been hampered by the limited number of effective genes and the complexity of the resistance mechanism in identified sources (Cuthbert *et al.* 2007). Sources of resistance to FHB are mostly restricted to Chinese hexaploid genotypes, such as Sumai3 and Wangshuibai (Mardi *et al.* 2005; Waldron *et al.* 1999) and, to a limited extent, Frontana from Brazil (Mardi *et al.* 2006). The lack of resistance sources in tetraploid wheat has limited the progress in durum wheat (*T. turgidum* L. var. *durum* Desf.) breeding for FHB resistance and has shifted the effort toward using the wild emmer wheat and wheat alien species (Oliver *et al.* 2005; Oliver *et al.* 2007).

The most effective quantitative trait loci (QTL) for Type II FHB resistance (resistance to disease spread within the spike) have been found on chromosome 3BS (*fhb1*) and chromosome 6BS (*fhb2*) of Sumai3-derived populations (Cuthbert *et al.* 2006, 2007). Otto *et al.* (2002) proposed a new source of resistance carried on the short arm of chromosome 3A from *T. diccocoides*, not located in a homeologous region as *fhb1* on 3BS. Another QTL for fungal penetration (Type I) and, to a lesser extent, spread (Type II) was consistently found on chromosome 5A (*Qfhs.ifa-5A*) (Buerstmayr *et al.* 2009; Buerstmayr *et al.* 2003). So far the QTL for FHB resistance have been identified on all of the wheat (mostly hexaploids) chromosomes except for 7D (Buerstmayr *et al.* 2009). As the major genetic effect of the FHB resistance genes is additive, it should be possible to accumulate different genes to enhance FHB resistance in wheat (Bai *et al.* 2000).

Association mapping utilizes linkage disequilibrium (LD) to discover marker/trait associations for a set of diverse germplasm or sets of inbred lines resulting from multiple crosses (Zhu *et al.* 2008). Association mapping analysis was initially developed for human linkage studies due to obvious limitation of structured populations derived from controlled crosses. Association mapping or LD mapping has been extended to plant studies, and many QTL have recently been identified and confirmed by means of this method (Agrama *et al.* 2007; Breseghello and Sorrells 2006; Casa *et al.* 2008; Christopher *et al.* 2007; Crossa *et al.* 2007; Maccaferri *et al.* 2011; Parisseaux and Bernardo 2004; Skøt *et al.* 2007; Stich *et al.* 2006; Tommasini *et al.* 2007). Association mapping is not only powerful in detecting QTL in natural populations or germplasm collections (Abdurakhmonov and Abdukarimov 2008), but it is also a good approach for detecting QTL in a routinely generated breeding program termed “*in silico* mapping” (Parisseaux and Bernardo 2004). Achieving success in association mapping depends on the separation of LD due to linkage or true association from other factors that make spurious associations (Malosetti *et al.* 2007; Stich and Melchinger 2009) and the statistical analysis that eliminates false positives (Kang *et al.* 2008). There are many advantages of association mapping in breeding populations compared with traditional QTL mapping, such as use of large populations with phenotypic data collected through multiple locations and years, diverse genetic backgrounds with multiple allele polymorphism, and availability of populations and phenotypic data (Parisseaux and Bernardo 2004).

By using breeding populations, the probability of having false positive LD due to the population structure and familial relatedness is increased (Myles *et al.* 2009; Zhu *et al.* 2008). This can be solved by using a linear regression model (Breseghello and Sorrells 2006) or a logistic regression model (Pritchard *et al.* 2000; Thornsberry *et al.* 2001) to correct for population structure. Principal component analysis (PCA) was used by Price *et al.* (2006) to account for subpopulation effects. By using these two methods, just limited events of relatedness that occur in a few axes of variation can be captured and removed from analysis. For example, with extended pedigrees where many of the individuals have a close relatedness (such as most breeding populations), a pairwise relatedness matrix called the kinship matrix (K) can be used to remove the false positive LDs due to the structure, selection, and admixture (Myles *et al.* 2009). Yu *et al.* (2006) proposed a linear mixed model to combine the outcome of population structure (Q matrix) with the marker-based K matrix, and they showed its power in reducing the number of false positives. This approach was successfully implemented in potato (Malosetti *et al.* 2007), Arabidopsis (Kang *et al.* 2008), maize (Weber *et al.* 2007), and wheat with minor modifications to improve the power of the mixed model (Stich *et al.* 2008) and to increase computational speed (Kang *et al.* 2008).

The North Dakota State University durum wheat breeding program has identified four tetraploid wheat sources of resistance from Tunisia, which were selected among a large number of lines evaluated over five repeated FHB trials. As the pedigree of these Tunisian lines shows no relation to Chinese genotypes, it is expected that they carry different alleles for resistance to FHB and could complement those loci. The objectives of our study were 1) to investigate the association of molecular markers with FHB resistance in different breeding populations derived from Tunisian lines; 2) to find the best model of association mapping analysis in the highly structured and related breeding populations; and 3) to compare the result of association mapping with classical QTL mapping analysis of one of the largest bi-parental populations in this analysis. The QTL identified in this study can be directly selected in the current breeding program utilizing these Tunisian-derived lines.

## Materials and Methods

### Plant material

**Figure 1:**
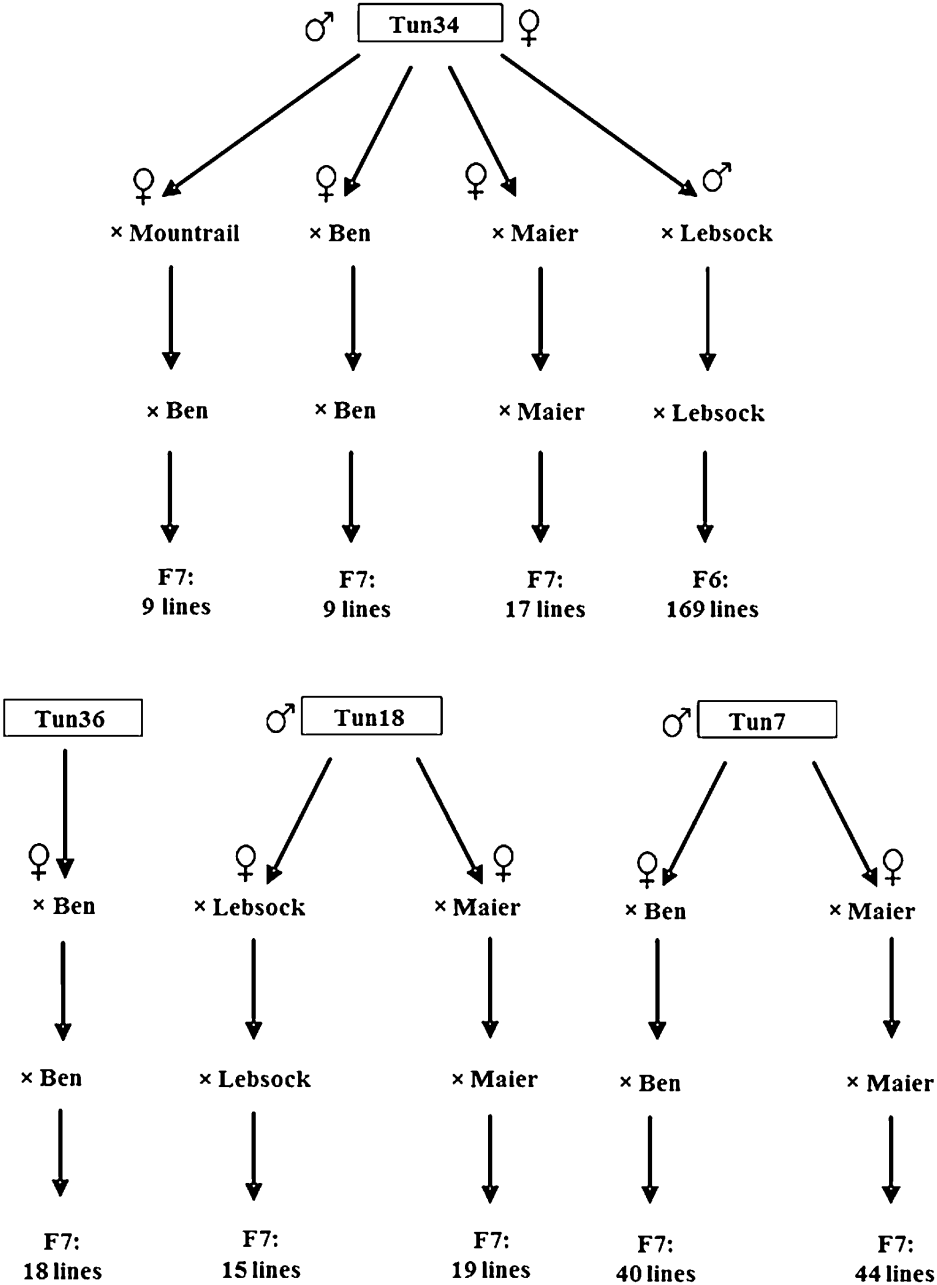
Pedigrees of durum wheat lines derived from FHB-resistant Tunisian lines.

A collection of backcross-derived advanced breeding lines consisting of resistant lines, susceptible sibs, and resistant sources were used in this study. A total of 171 BC_1_F_7_ and 169 BC_1_F_6_ lines derived from multiple crosses of four Tunisian sources (Tun7, Tun18, Tun34, Tun36) with durum cultivars ‘Ben’ (Elias and Miller 1998), ‘Maier’ (Elias and Miller 2000a), ‘Lebsock’ (Elias *et al.* 2001), and ‘Mountrail’ (Elias and Miller 2000b) were used for association mapping analysis (Figure 1).

### FHB screening

Three different isolates of *Fusarium graminearum* (R010, R1267, and R1322) were cultured and prepared separately. A mixture suspension of these three isolates was prepared (50,000 conidiospores per milliliter) just prior to inoculation. A 10 µl droplet of the conidial suspension was injected into one of the florets in the middle of spike on each plant. The parents and RILs were screened in the greenhouse for two seasons during 2006 and 2007 for Type II resistance to FHB by the previously described method (Stack *et al.* 2002). The parents were planted in randomized complete block design (RCBD) with three and six replicates in 2006 and 2007, respectively, while progenies were planted in RCBD with two replicates in both years. Sumai3 and ND2710 (Frohberg *et al.* 2004), which are both hexaploid wheat, were used as controls for resistance, and D87450 (durum wheat) was used as susceptible check. The FHB severity was scored using 0 to 100% scale by counting the infected spikelets divided by the number of the total spikelets (infection rate) in a spike. To collect the phenotypic data, the infection rate from two inoculated spikelets per plant was reported, and the average of those values was used as the data point. (Data is provided in supporting information, File S2.)

### Genotyping

The DNA extraction was performed on a bulk of one leaf from at least 10 three-week-old plants per line following the methods described by Guidet *et al.* (1991) with minor modifications. The concentration of the DNA was measured by NanoDrop 2000 **(**Thermo Fisher Scientific, FL). The samples were diluted to the concentration of 100 ng/μl, and 20 μl of the samples were sent for DArT markers analysis by Triticarte Pty. Ltd., as described by Akbari *et al.* (2006). A total of 2300 DArT markers, distributed across the entire wheat genome, were screened on the entire population. (Data is provided in File S2.)

### Genetic mapping

The polymorphic markers for the population derived from the crosses of Tun34 and ‘Lebsock’ were analyzed using JoinMap 4.0 (Van Ooijen 2006). Markers were assembled into linkage groups at likelihood ratio statistic (LOD) ≥ 3.0 and assembled into a consensus map. Markers showing highly distorted segregation ratios (*P* < 0.005) were excluded from map assembly. The Kosambi mapping function (Kosambi 1943) was used to convert recombination frequencies into centimorgan map distances.

### Identification of QTL for FHB resistance in Tun34×Lebsock population

QTL analysis was performed by Kruskal-Wallis rank-sum test and interval mapping (Lander and Botstein 1989) on the genome using all 169 BC_1_F_6_ lines. Thereafter, multiple QTL mapping (MQM) on chromosome 5B (Jansen 1993; Jansen and Stam 1994) was carried out, where marker *wPt-7279* and all markers on chromosome 2A were considered as cofactors. The significant threshold LOD score (*P* = 0.05) for detection of QTL on the whole genome was determined by 1000 permutations tests (Churchill and Doerge 1994).

### Association mapping

To find the association between the genetic markers and resistance to FHB, 10 different models previously discussed by Stich *et al.* (2008) were tested to correct the structure and the coancestry relatedness in the entire population (see File S1). FastPHASE (Scheet and Stephens 2006) was used to impute missing data with default settings. Further, PowerMarker (Liu and Muse 2005) was used to find minor allele frequencies (MAF). Markers with MAF < 0.05 were removed from analysis.

The best K_T_, QK_T_, and PK_T_ matrices were obtained using the lowest mean square difference (MSD) value among the 20 T value comparisons. The expected *P* values used for MSD calculation are obtained by dividing the rank of an observed *P* value with the total number of markers (Stich *et al.* 2008). We selected the best models, considering the lowest MSD between observed and expected *P* values of all marker loci and percentage of observations below nominal level (α = 0.05) in a *P*(expected)-*P*(observed) plot (see File S1).

For the selected model(s), positive false discovery rate (pFDR; Q values) were calculated (Storey 2002) using the PROC MULTTEST procedure in Statistical Analysis System (SAS 9.2) software. The markers associated with the FHB trait are based on a cutoff criteria of *P* < 0.05 and Q value < 0.1 (Weber *et al.* 2007). For the significant markers, the phenotypic variation (*R^2^*) was calculated using a simple regression equation.

### Linkage disequilibrium

For markers mapped in the Tun34×Lebsock population with a known location on the genetic map, the LD coefficient (*r*^2^) was plotted against genetic distance, and locally weighed polynomial regression (LOESS)–based fitting curves were used to infer the decay of LD as described in Breseghello and Sorrells (2006) and Maccaferri *et al.* (2011). The 95th percentile of the distribution of unlinked markers (markers on different chromosomes) was used to set the critical *r*^2^ value.

## Results

### Genetic analysis and heritability of Tunisian-derivative populations for FHB

The pedigree of 323 Tunisian-derived backcross inbred lines (BIL) and nine triple-cross inbred lines used for this study are shown in Figure 1. These lines were selected in the field for their FHB resistance and agronomic performance for cultivar development. However, they were also evaluated in the greenhouse for Type II resistance by the single-floret inoculation method described by Stack *et al.* (2002). There were statistically significant differences in FHB Type II disease severity between genotypes and also between the seasons (Table S1). The effects of two greenhouse seasons were not significant on the cultivars’ infection reaction. The same scenario was seen for the entire population, as the correlation between the two season was very high (*r* = 0.91; *P* < 0.0001), and the effect of cultivar × season was not significant (α = 0.05). Therefore, FHB mean score values across the seasons were used as reliable data points in the analysis. Broad sense heritability was estimated to be 81% by calculation based on ANOVA (Bai *et al.* 2000).

**Figure 2:**
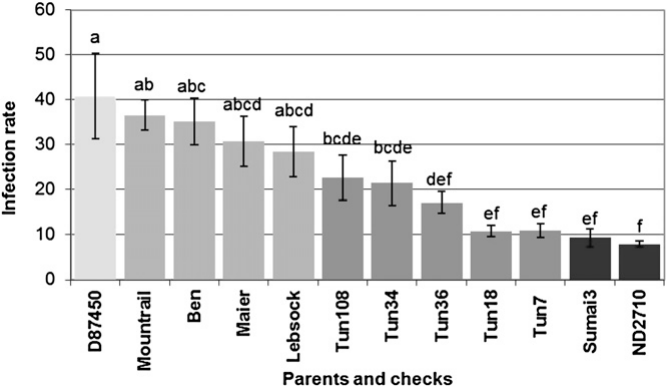
The average of infection rate for susceptible durum cultivars and the Tunisian resistant sources derived from nine plants (two spikes in each plant) planted in two seasons in 2006 and 2007. D87450 was used as the susceptible check, and Sumai3 and ND2710 were used as the resistant controls. The letters on top of each column indicates the Duncan grouping of means at the probability level of 0.05.

The results show that Tun7 and Tun18 have resistance comparable to the Chinese hexaploid source Sumai3 (Figure 2). Although Tun34, Tun36, and Tun108 show better resistance levels compared with common durum cultivars, their level of resistance is not statistically different from ‘Lebsock’ and ‘Maier.’ The Tun34×Lebsock cross had the largest population size (169 lines) and, therefore, was used for genetic mapping of the markers and classical QTL analysis. The progenies from this cross showed transgressive segregation for resistance to FHB. Nearly 8.5% of the progenies expressed higher levels of resistance to FHB, whereas 53% were more susceptible compared with the parents (Figure S1). Most of the other crosses (but not Tun18×Lebsock or Tun34×Ben) showed transgressive segregation for FHB resistance. Most of the crosses with ‘Maier’ produced plants with more resistance than both parents even in Tun7 and Tun18 crosses. These crosses produced progenies (1 out of 44 for Tun7×Maier population and 1 out of 18 for Tun18×Maier population) with the same resistance as Sumai3 and spring wheat resistant breeding line ND2710.

**Figure 3:**
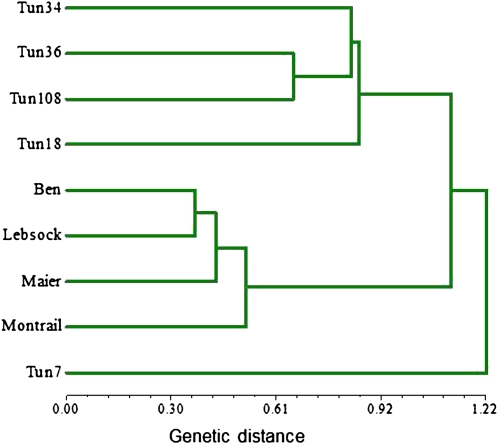
Genetic distance (D = −LN J; J = Jaccard coefficient) dendrogram of Tunisian sources of FHB resistance and durum wheat cultivars based on 537 DArT markers scanning the whole genome.

All the parents and progenies depicted in Figure 1 were genotyped using the DArT marker system. This system enabled scanning the entire genome to identify segments that carry FHB resistance genes (QTL). In DArT analysis, 2300 markers were used, of which 23% were polymorphic between the parents. About 8% of the polymorphic markers were present in all the Tunisian lines but absent in the susceptible cultivars. Cluster analysis of the polymorphic DArT markers revealed three distinct groups (Figure 3). The Tun7 line was in a separate group from the other two, and all other Tunisian lines were clustered in a separate group from susceptible durum cultivars.

### Construction of genetic map

**Figure 4:**
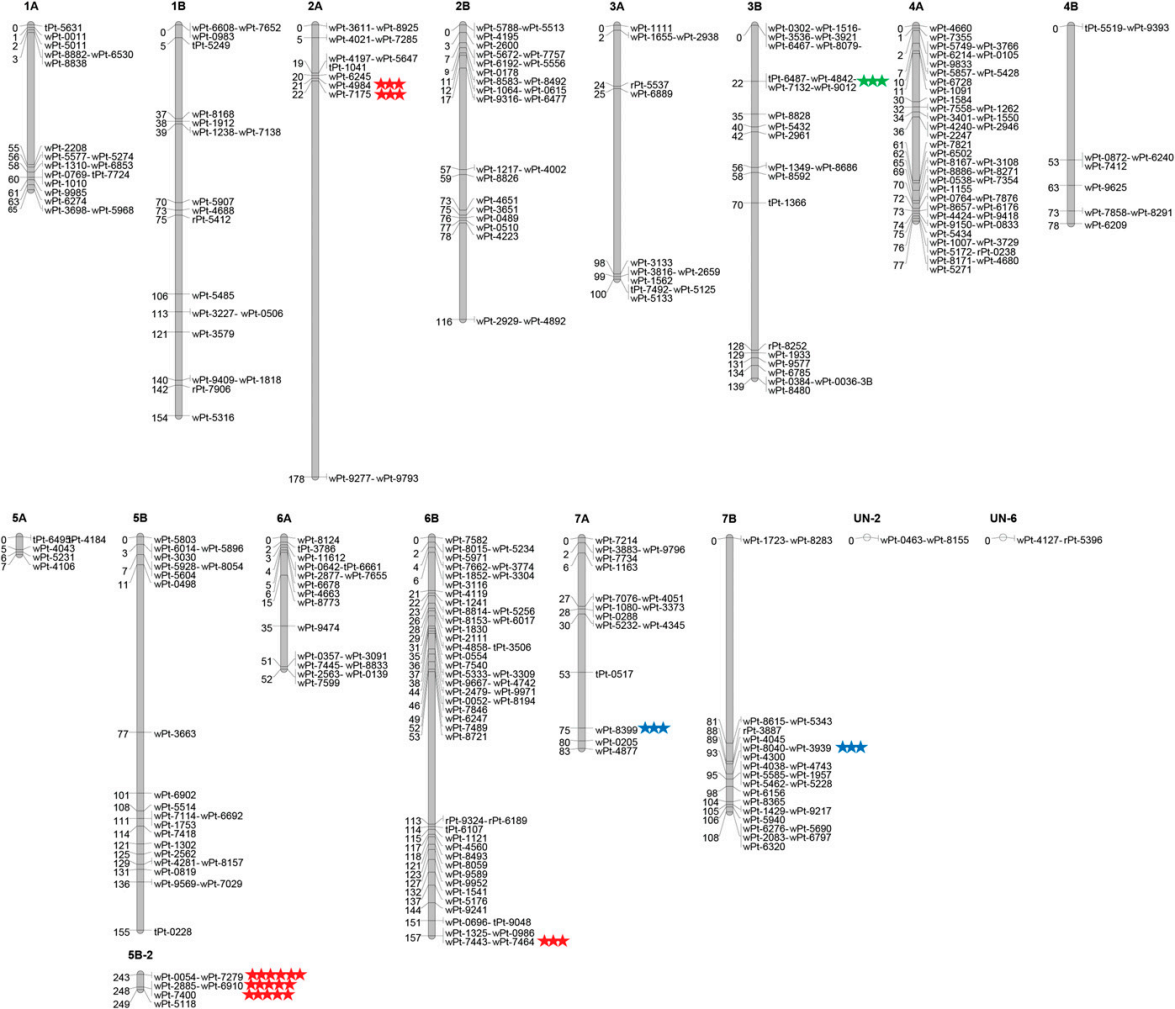
Genetic maps and approximated location of the QTL on the chromosome arms of Tun34×Lebsock population (units are cM). The associated markers to Type II FHB resistance found by both QTL and mixed model association mapping analysis are indicated by red stars. The markers revealed only by Kruskal-Wallis test are in blue and the one just found by linear mixed model analysis is in green. However, the only major QTL confirmed in this study is *Qfhs.ndsu-5BL*.

A genetic linkage map based on the population of Tun34×Lebsock was constructed. Out of 2300 DArT markers, 15% (379) were polymorphic after screening Tun34 and Lebsock parents. Genotypic data were incorporated into different linkage groups using the JoinMap 4.0 software (Van Ooijen 2006). The observed segregation ratio was compared with expected ratios for all markers using the chi-square goodness-of-fit test. Results indicated the segregation distortion of 5.2% (*P* < 0.01) for this population. To eliminate the bias effect of genetic mapping due to distorted loci, highly distorted loci (*P* < 0.005) were excluded from the analysis before mapping. Out of 379 markers, 359 were assigned (LOD ≥ 3.0) into 44 linkage groups, with the minimum number of three markers per group. As is illustrated in Figure 4, almost all of the linkage groups (except 2) could be assigned to durum wheat chromosomes by alignment to previously published maps (Mantovani *et al.* 2008; Peleg *et al.* 2008; Semagn *et al.* 2006). The location of the DArT markers corresponded well to previously published durum wheat DArT maps with a few exceptions. As the 359 markers couldn’t cover the whole genome, there were low-coverage regions on chromosomes 2A, 3A, 4A, 5A, 4B, 5B, and 7B and large gaps introduced to connect separate linkage groups on chromosomes 2A, 7B, and 5B. Overall, this map provided nearly 75% coverage of the genome compared with published maps.

### Genomic regions associated with FHB resistance

The phenotypic and genotypic data from the Tun34×Lebsock population were analyzed by a nonparametric genomic scan based on the Kruskal-Wallis rank-sum test (*P* ≤ 0.001) using the MapQTL5 software (Van Ooijen 2004) to identify genetic markers associated with putative QTL for FHB resistance. Results shown in Table 1 revealed five regions associated with FHB resistance, located on chromosomes 5B, 2A, 6B, 7A, and 7B. A region on chromosome arm 5BL (4 cM interval) showed the highest K score and an increase in FHB resistance due to alleles from Lebsock parent. Other QTL identified by this method have a lower K score (Table 1). Following the method described above, interval mapping (IM) was performed on the whole genome. The results of IM revealed presence of a significant QTL (LOD = 4.5) on chromosome 5B accounting for 11.8% of phenotypic variation (14.6% of genetic variation) for FHB. The significant threshold (LOD = 3.4) was based on a permutation test implemented as described in *Material and Methods*.

**Table 1:** Genomic regions associated with Fusarium head blight resistance in Tun34×Lebsock BC_1_F_6_ population

Group	Position (cM)	Locus	K*^a^*	*P*
5B-2	243-247	wPt-0054	17.115	10^−6^
5B-2	243-247	wPt-7279	15.188	10^−6^
5B-2	243-247	wPt-2885	14.543	10^−5^
5B-2	243-247	wPt-6910	13.205	10^−5^
5B-2	243-247	wPt-7400	12.652	10^−3^
2A	20-22	wPt-7175	8.037	10^−3^
2A	20-22	wPt-4984	6.823	10^−2^
6B	156	wPt-7443	6.93	10^−2^
7A	75	wPt-8399	7.287	10^−2^
7B	93	wPt-8040	7.463	10^−2^
7B	93	wPt-3939	6.745	10^−2^
7B	93	wPt-4300	7.584	10^−2^

^a^ Kruskal-Wallis test statistic (df = 1).

To further study the major QTL on chromosome 5B, multiple QTL mapping (MQM) analysis was performed on this population. The phenotypic value explained by this QTL when *wPt-1723* (7B), *rPt-3887* (7B), and *wPt-0054* (5B) were chosen as cofactors increased to 14.7% (18.1% of the genetic variation). Although the position of the QTL did not change, LOD score increased to 6.1.

### Linkage disequilibrium analysis

**Figure 5:**
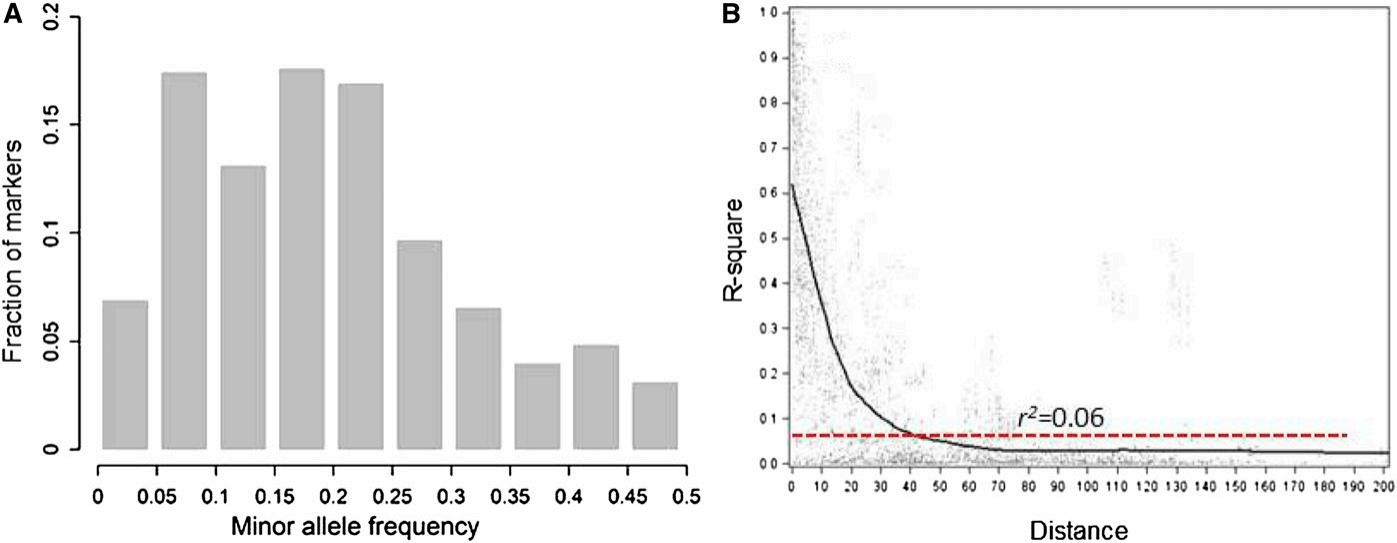
(A) The frequencies of minor alleles in the populations are maximized at 0.2 due to the effect of selection. (B) The estimates of *r*^2^ *vs.* the genetic distances of the markers according to Tun34×Lebsock genetic map. The LD decay is around 40 cM by considering the critical value of 0.06.

For LD analysis, 537 polymorphic markers were selected on the whole population of nine different crosses. As the frequencies of the alleles play an important role in LD analysis, we eliminated the alleles with minor frequencies of less than 0.05 (Figure 5A). As shown in Figure 5A, the frequency of the alleles shifted from 0.5 to 0.2 due to several rounds of selection in the breeding populations. The LD decay graph (Figure 5B) shows the LD decreased with increasing genetic map distance between marker loci. In this graph, syntenic *r^2^* (estimated LD for the loci on the same chromosome) was plotted against map distance. The 95th percentile in the distributions of the estimated LD of unlinked loci (*r^2^* = 0.06) was used to estimate the extent of LD across the genome according to Breseghello and Sorrells (2006). The intersection of the LOESS fitting curve at this critical LD threshold was estimated to be 40 cM. As the LD breakdown depends on the number, relatedness, and mating system of the lines, this high degree of LD is not unexpected considering the selection pressure for FHB resistance and backcrossing to generate the populations (Abdurakhmonov and Abdukarimov 2008; Flint-Garcia *et al.* 2003).

### Association mapping analysis

The association mapping was implemented not only on the entire data set but also for the lines derived from crosses of the same Tunisian resistant source. This could increase the frequency of alleles from the resistance parents in the population if they carry different alleles for FHB resistance. By dividing the data into different panels, the power of association mapping on population under 100 lines could be tested. The comparison between association mapping on the Tun34 panel and the classical QTL mapping results on Tun 34×Lebsock was also possible in our experiment.

For each association method, the high MSD between observed and expected *P* values (uniform distribution) of all marker loci indicates that the empirical Type I error rate of these approaches is considerably higher than the nominal α-level (Stich *et al.* 2008). Therefore, MSD between observed and expected *P* values calculated for three different association mapping models (K_T_, QK_T_, and PK_T_) for all 20 measures for T value was used to find the best T value for each model (see File S1).

The comparisons of all 10 models are summarized for the three different panels in Table S2. For the entire data, the MSD value of the K_T(0.65)_ and QK_T(0.65)_ was the lowest. Since the *P* values are assumed to be under uniform distribution (Yu *et al.* 2006), models that have a higher number of *P* values under a critical limit are usually non-uniform. The Naïve model has ∼11% of the observations under 5% threshold levels, while the two selected models (K_T_ and QK_T_), have only 5% of the observations under the 5% threshold level. In Tun34 panel PK_T(0.30)_ had the lowest MSD (0.0003), and only 6% of the observations were under the 5% threshold level. The performance of this model is better than Naïve model with MSD of 0.32. For the Tun7 panel, however, PK_T(0.55)_ had the minimum MSD value (Table S2).

For the entire data set, a union output of the two different models (K_T_ and QK_T_) showed that markers from 1B, 2A, 3A, 3B, 4A, 5B, 6A, 6B, 7A, and 7B are associated (*P* < 0.05) with FHB (Table 2). Of these 35 markers, association of 5 markers was significant after correcting for multiple testing using pFDR criterion (Q value < 0.1). All of these markers were from the same QTL located on 5BL. The other QTL found in this study were not confirmed by Q value less than 0.1, although the QTL from 3AS, 3BS, and 6BL seems promising as the pFDR criterion is close to significance. Twenty markers out of 35 could be mapped in Tun34×Lebsock population (Table 2). The mapping information of the rest of the markers in Table 2 was extracted from a consensus map version 4.0 released by Triticarte Pty. Ltd.

**Table 2:** Associated markers to FHB Type II resistance

Marker	Chromosome	cM	P-value	pFDR	*R^2^*	MAF
wPt-1876	1B	29	0.031	0.793	0.010	0.27
wPt-9369	3A	45	0.002	0.174	0.020	0.08
wPt-7992	3A	59	0.010	0.473	0.014	0.10
wPt-6854	3A	44	0.010	0.473	0.013	0.10
wPt-2305	5B	24	0.030	0.793	0.019	0.10
wPt-7663	6A	23	0.018	0.235	0.024	0.21
wPt-8554	6B	68	0.023	0.235	0.008	0.13
wPt-2162	6B	107	0.002	0.131	0.012	0.08
wPt-9256	6B	115	0.016	0.235	0.003	0.09
wPt-4831	7A	122	0.032	0.793	0.016	0.13
wPt-4025	7B	146	0.029	0.235	0.025	0.09
wPt-8981	7B	149	0.014	0.561	0.028	0.24
wPt-9665	7B	149	0.021	0.667	0.026	0.24
wPt-4533	2A	18	0.033	0.235	0.002	0.06
**wPt-4021**	2A	5	0.020	0.667	0.017	0.15
**wPt-4984**	2A	21	0.039	0.812	0.027	0.26
**tPt-1041**	2A	19	0.042	0.812	0.026	0.24
**wPt-7285**	2A	5	0.049	0.812	0.014	0.13
**tPt-6487**	3B	22	0.048	0.812	0.011	0.20
**wPt-6467**	3B	0	0.002	0.188	0.004	0.32
**wPt-4842**	3B	22	0.014	0.235	0.009	0.20
**wPt-5434**	4A	75	0.044	0.235	0.023	0.17
**wPt-0054**	5B	243	0.000	0.028	0.061	0.14
**wPt-2885**	5B	248	0.000	0.039	0.050	0.11
**wPt-7400**	5B	248	0.000	0.040	0.045	0.11
**wPt-5118**	5B	249	0.003	0.191	0.039	0.13
**wPt-6910**	5B	248	0.000	0.064	0.058	0.12
**wPt-7279**	5B	243	0.001	0.098	0.055	0.14
**wPt-1302**	5B	34	0.041	0.235	0.006	0.34
**wPt-1121**	6B	115	0.040	0.812	0.026	0.13
**tPt-6107**	6B	114	0.044	0.812	0.026	0.13
**tPt-9048**	6B	151	0.044	0.812	0.020	0.23
**wPt-8059**	6B	121	0.023	0.235	0.023	0.20
**wPt-9241**	6B	144	0.043	0.235	0.019	0.26

Values are based on the union output of the K_T_ and QK_T_ mixed model analysis of 537 markers in 340 RILs derived from nine different crosses. The positive false discovery rate (pFDR) test only confirmed the association of the 5BL markers to FHB resistance. *R^2^* is calculated using a simple regression model. The position of the bolded markers are found through Tun34×Lebsock population, and the other positions are based on the consensus map version 4.0 released by Triticarte Pty. Ltd. Minimum allele frequency (MAF) of each marker is reported in the last column.

For Tun34 panel, 24 markers had significant association with FHB resistance at *P* < 0.05 using the PK_T(0.30)_ model. However, none of the associated markers had an acceptable pFDR (Q value < 0.1). By increasing the threshold for pFDR to Q value < 0.15, the same associated markers to FHB resistance can be found as the entire data set such as *wPt-2885*, *wPt-6910*, *wPt-7400*, and *wPt-0054* from 5BL. By increasing the threshold, a marker from chromosome 3B (*tpt-6487*) can be considered as having a significant association to FHB resistance with a *P* value < 0.0006 and a Q value < 0.15. Although 13 markers had significant association to FHB resistance in Tun7 panel, none of them had an acceptable amount of pFDR, and all are considered as false positives in the analysis.

## Discussion

There are limited sources of resistance to FHB in wheat, especially in durum or pasta wheat. Owing to this limitation, tremendous efforts have been made to introduce new sources of resistance from wild tetraploids, such as emmer (*T. diccoccum*), Persian (*T. cathalicum*), and Polish (*T. polanicum*), and from common wheat sources, such as Sumai3, to durum wheat with limited success (Garvin *et al.* 2009; Kumar *et al.* 2007). Transferring FHB resistance from other alien species, such as *Lophopyrum elongatum*, were reported to have produced durum lines with FHB resistance (Jauhar and Peterson 2009), but successful use of these lines carrying alien chromosomes in breeding programs has been a challenge. Two FHB-resistant Tunisian lines (Tun7 and Tum18) were found with promising levels of resistance to FHB Type II and were integrated into the North Dakota State University (NDSU) durum wheat breeding program. These two lines both show consistent low infection rates of about 10% comparable to the Sumai3, which is the most widely used source of resistance in hexaploid wheat (Figure 2). These two genotypes are also believed to represent different genetic backgrounds based on the genotyping results (Figure 3). This increases the likelihood of having different genes/alleles for Type II FHB resistance derived from these sources.

Somers *et al.* (2006) crossed an accession of *T. carthalicum*, showing a moderate Type II resistance to FHB, with a durum wheat cultivar ‘Strongfield’ to introduce new QTL into a cultivated background. They observed transgressive segregation for FHB resistance in their population, with a few lines carrying QTL from both parents being more resistant than either parent. However, none of these lines have been released as a new cultivar. In this study, Tun34×Lebsock cross resulted in a population with transgressive segregation for FHB resistance. Both parents had an infection rate of about 20 to 30%, but a portion of the population (∼8.5%) were found to have within 10 to 20% range of Type II disease severity. This may be due to the fact that ‘Lebsock’ is also moderately resistant and many minor genes for FHB resistance would be segregating in this population. Other subpopulations also show transgressive segregation, especially those derived from ‘Maier’, where some progenies had resistance levels similar to that of Sumai3 and ND2710 (a resistant spring wheat line derived from Sumai3). If these lines prove agronomically suitable, they can be released as new resistant cultivars.

An advantage of doing association mapping in a breeding population is to investigate the associated genes to the trait of interest and track them in the process of breeding and selection. Large LD blocks are common in most breeding populations, which can be reduced by backcrossing with the cultivated parent. Having the associated markers in the same background provides a valuable tool to perform several backcrosses and reduce the LD blocks while tracking the resistance genes in the progenies by marker-assisted selection. The LD decay plot of *r^2^* values *vs.* genetic distances between all markers across the genome showed that the LD extends up to 40 cM (Figure 5B). A number of factors can explain the large LD blocks observed in this study, such as mating system (self-pollination), population structure, relatedness (kinship), small number of population founders, admixture, epistasis, and selection (Abdurakhmonov and Abdukarimov 2008). There are different reports for LD decays in different self-pollinated crops. The extent of LD was 10 to 50 cM in 953 cultivated barley accessions (Malysheva-Otto *et al.* 2006) and 10 to 20 cM in 43 US elite wheat cultivars representing seven market classes (Chao *et al.* 2007). Crossa *et al.* (2007) mapped 318 DArT markers in two subpopulations of five CIMMYT elite spring wheat yield trials and found LD decay around 40 cM. Therefore, finding large LD blocks in this study was not unexpected for the small number of pedigrees, selection pressure placed on these lines for various characteristics by the breeding program, and the backcrossing scheme used to generate the populations. However, using a collection of diverse elite accessions could reduce the LD blocks, as was seen by Maccaferri *et al.* (2011) in their study. Extending LD in self-pollinated crops, especially in backcross-derived inbred lines from a limited number of crosses, reduces the chance of finding tightly linked markers to the QTL of interest but eliminates the necessity of applying a large number of markers on the population. Thus, in these types of populations, finding the QTL might be possible by assaying just a few Single Sequence Repeats (SSR) markers 10 to 20 cM apart on each chromosome.

The result of our association mapping analysis showed the QK_T_ and the K_T_ method were the best for finding the QTL in highly structured and related breeding populations. In the subpopulations, such as Tun34 and Tun7 crosses, the linear mixed model that takes the structure and kinship into account was found to be the best. The PK_T_ was better than the QK_T_ method as small populations under selection pressure deviated from the Hardy-Weinberg equilibrium, which may not affect PCA but can affect structure matrix (Q). The results show that replacing the K matrix in the QK and PK models with K_T_ improves the power of the association analysis by 2-fold. Stich *et al.* (2008) also proposed that the mixed model approach using a kinship matrix estimated by REML is better than marker-based kinship estimates underlying the studies of Yu *et al.* (2006). Despite the availability of pedigree information, the pedigree method (G) did not perform the same as other methods and was only better than Naïve model.

The QTL analysis on Tun34×Lebsock population identified a major QTL, previously not reported in durum wheat, on chromosome 5BL (hereby designated as *Qfhs.ndsu-5BL*) explaining between 14.6 and 18.1% of genetic variation (H^2^ = 0.81). Classical QTL analysis corroborated the result of association mapping for this QTL. This further indicates that the mixed models association analysis applied here identified the major QTL with high allele frequency in the population. The highly structured population after several rounds of selection may reduce frequency of the allele with minor effects, especially when those selections are in favor of agronomics traits and not only FHB resistance. These would reduce the power of genome-wide association mapping to detect those QTL (Long and Langley 1999). In the case of FHB resistance where multiple genes with moderate effects are involved, genome-wide association mapping would fail to find these genes, especially when they are present in only one or a few subpopulations. Despite this drawback, potential QTL on chromosomes 3AS, 3BS, and 6BL by LD analysis were also identified but could not be confirmed by the pFDR test. Although an adjustment for multiple comparisons seems to be necessary for association mapping analysis to eliminate the false positives (Sabatti *et al.* 2003), a high stringent FDR threshold can lead to unexpected false negative errors as well (Park and Mori 2010).

Focusing on at the Tun34 panel alone, similar pFDR for *wPt-0054* (5BL) and *tPt-6487* (3BS) is noted. In the entire panel, the Q value for *wPt-5118* from 5BL is even slightly higher than the Q value for *wPt-6467* from 3BS (Table 2). Therefore, it is very likely that another QTL for FHB resistance on chromosome 3BS exists in these populations. This was not true for other significant QTL, such as 1B, 2A, 3A, 4A, 6B, 7A, or 7B, which could not be confirmed by pFDR. As the associated markers from 3AS and 7BL regions were monomorphic in the Tun34×Lebsock population, their allele frequencies could not be determined, and the Q value was nonsignificant. Therefore *wPt-9369* from 3AS or *wPt-4025* from 7BL could be interesting markers for future FHB studies in Tunisian lines. The locations of these QTL are in the approximate location of *Qfhs.ndsu-3AS* found in *T. turgidum* L. var. *dicoccoides* (Otto *et al.* 2002) and the 7BL QTL found for Type II resistance in spring wheat population (Yang *et al.* 2005). Surprisingly, given the lack of connection between our material and the Chinese hexaploid wheat resistance sources, the location of *tPt-6487* (3BS) is in the approximate location of the major gene for FHB resistance *fhb1* identified in later material (Cuthbert *et al.* 2006).

There is also the possibility of having a QTL influencing FHB resistance (*tPt-1041* and *wPt-4984*) on chromosome 2A from the Kruskal-Wallis test conducted in our classical QTL and association mapping analyses but not confirmed by pFDR. This region is in approximate location of the 2A QTL found by Garvin *et al.* (2009), which may mask the magnitude of the resistance from 5BL QTL. Recently, Garvin *et al.* (2009) proposed the presence of a genomic region on chromosome 2A of wild emmer wheat that increases the FHB severity in durum wheat. Here the Tun18 and Tun7 both carry the 5BL and 3BL resistance QTL and seem not to carry the 2A susceptibility QTL (Figure 4). Therefore, both show good levels of Type II resistance (Figure 2). On the other hand, Tun34 and Tun36 both have only the 3BS resistance QTL but potentially carry the 2A susceptibility QTL and show a moderate level of FHB resistance (Figure 2). The majority of durum cultivars also show similar genotype for this susceptibility region. Thus, this finding not only further corroborates the result of Garvin *et al.* (2009) but also identifies the path for developing more resistant durum varieties. The influence of *Qfhs.ndsu-3AS* in the *T. dicoccoides* accession ‘FA-15-3′ (Syn. ‘Israel A’) was not revealed until elimination of the suppression activity of the 2A QTL by using the chromosome substitution lines (Stack *et al.* 2002). The effect of the suppressor gene is so adverse that, despite the presence of the 3A QTL for FHB resistance, the overall phenotypic reaction of FA-15-3 is highly susceptible (Garvin *et al.* 2009). Pyramiding the QTL for FHB resistance in durum wheat background would not be helpful unless we have counter selection against 2A QTL.

This study illustrates the advantage of QTL mapping in validating the association mapping result as recently proposed by Brachi *et al.* (2010) in Arabidopsis. The presence of 5BL QTL was confirmed by both association and classical QTL mapping. The presence of 3BS QTL in the populations analyzed here needs further confirmation by either increasing the size of the populations or developing validation material. Controlling the false positives and negatives for the highly structured, advanced population studied here would always be a challenge, especially when there is selection in favor of some other agronomic traits. Detecting QTL with minor effects, low frequencies, and allelic interaction (*i.e.* epistasis) would be a challenge in association mapping (Hall *et al.* 2010). The analysis presented here indicates that association mapping of complicated traits inherited quantitatively and influenced by environment, such as resistance to FHB, in highly structured breeding populations is possible. The number of false positives was very low in our association analysis. This also indicates that the linear mixed model considering the structure (Q or P) and the kinship matrix estimated by REML (K_T_) would be good models for association mapping in a mixture of wheat populations from different breeding programs.

The possibility exists of having false negative associations when dealing with a trait of complex inheritance controlled by multiple genes each having moderate effects. This can be accounted for by increasing the number of populations resulting from each cross. Zhu *et al.* (2008) recommend a large sample size (more than 250) to obtain high power to detect genetic effect of moderate size. We recommend association mapping with multiple subpopulations having more than 100 lines (in F_5_ or F_6_ generation) before any other selection, except for the trait of interest, is placed on them. Working with different subpopulations derived from multiple resistance sources would increase the probability of finding different resistance QTL in a single experiment. Ten out of the 22 QTL found so far for FHB resistance are associated to plant height (Buerstmayr *et al.* 2009). Therefore selection in the favor of short plants would reduce the frequency of the alleles for FHB resistance in breeding populations by about 45%.

The results of our study indicate the power of genome-wide association mapping in finding QTL for FHB resistance in highly structured breeding populations. The *Qfhs.ndsu-5BL* found in this study was further validated by classical QTL mapping, emphasizing again the effectiveness of mixed model association mapping for a complex trait such as FHB resistance. Replacing the K matrix in the QK and PK models with K_T_ improved the power of QTL detection in backcross-derived inbred lines, which was also indicated by Stich *et al.* (2008) in soft winter wheat inbred lines. The 3BS QTL found in this study, which has been located in the approximate location of *fhb1* gene, would eliminate the need to introduce the gene from hexaploid Chinese sources, which have not been successful in developing released varieties. Additionally, the confirmation of a 2A QTL for susceptibility (or suppressor of resistant) to FHB emphasizes the need to devise a better strategy for improving FHB resistance in durum wheat by elimination of this locus.

## Supplementary Material

Supporting Information
