# Supplementary material for: Mixed Model Association Mapping for Fusarium Head Blight Resistance in Tunisian-Derived Durum Wheat Populations
Source: G3 (Bethesda). 2011 Aug 1;1(3):209–18. doi: 10.1534/g3.111.000489 (PMC3276138; doi:10.1534/g3.111.000489)
Supplement: Supporting Information [file supp_1_3_209__index.html]

Supporting Information 

# Mixed Model Association Mapping for Fusarium Head Blight Resistance in Tunisian-Derived Durum Wheat Populations

## Supporting Information for Ghavami *et al.*, 2011

**Files in this Data Supplement:**

- Supporting Information - Figure S1, Files S1 and S2, and Tables S1 and S2 (PDF, 760 KB)
- Figure S1 - Frequency distribution of FHB severity among 169 BC1F6 wheat RILs of the Tun 34�Lebsock cross measured in the two greenhouse seasons in 2006 and 2007. (PDF, 64 KB)
- Table S1 - Analysis of variance of infection rate for Type II FHB resistance measured in greenhouse in two seasons in 2006 and 2007 (PDF, 44 KB)
- Table S2 - Mean of the squared differences (MSD) between observed and expected *P*-values for various association mapping models (PDF, 56 KB)
- File S1 - Association Model Testing (PDF, 556 KB)
- File S2 - Supporting Data (Microsoft Excel, .xlsx, 652 KB)
