# Supplementary material for: Mixed Model Association Mapping for Fusarium Head Blight Resistance in Tunisian-Derived Durum Wheat Populations
Source: G3 (Bethesda). 2011 Aug 1;1(3):209–18. doi: 10.1534/g3.111.000489 (PMC3276138; doi:10.1534/g3.111.000489)
Supplement: Supporting Information [file supp_1.3.209_000489SI.pdf]

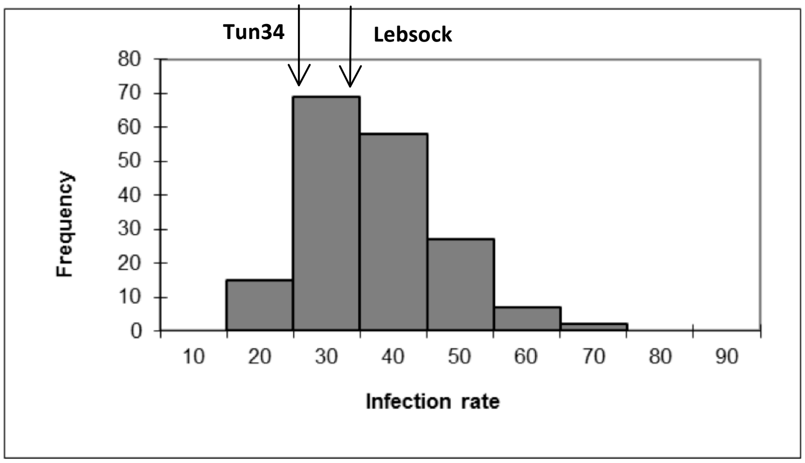

Figure S1 Frequency distribution of FHB severity among 169 BC_1F_6 wheat RILs of the Tun 34×Lebsock cross measured in the two greenhouse seasons in 2006 and 2007.

File S1
Association Model Testing

METHODS:

Ten different statistical models used for the association analysis are summarized in Table 1 as described by STICH *et al.* (2008). The statistical formula used for the models was:

$$y = X\alpha + Q\beta + Iv + \varepsilon,$$

Where y is a vector of phenotypic values, β is a vector of fixed effects regarding population structure, α is the fixed effect for the candidate marker, *v* is a vector of the random effects pertaining to co-ancestry, and ε is a vector of residuals. *Q* is a population structure matrix that can be replaced by "*P*", which is a matrix of the significant principal component vectors that explain 50% of variation. *X* is the vector of genotypes at the candidate marker, and *I* is an identity matrix. The variances of the random effects are assumed to be $\mathrm{Var}(v) = 2KV_g$ and $\mathrm{Var}(e) = IV_R$, where *K* is the kinship matrix , V_g the genetic variance, and V_R the residual variance (YU *et al.* 2006).

Population structure was calculated using the software STRUCTURE (PRITCHARD *et al.* 2000). The output is a matrix with the columns equal to the number of subpopulations, with the probability of membership of each individual in a subpopulation. We analyzed the number of subpopulations between 1 and 10 for a total of 5 replicates. For each run, a 100,000 iterations were used as burn-in, followed by 500,000 Markove chain Monte Carlo iterations. The optimum number of sub-populations is determined using the Wilcoxon two sample test described in WANG et. al. (2008) implemented using PROC NPAR1WAY in SAS 9.2® (SAS Institute, Cary, NC). We sequentially compared the posterior probabilities from all the 10 runs of a given *k,* with those of probabilities from the run with a *k* value one larger (*k*=1 vs. *k* =2; *k* =2 vs. *k* =3 and so on). The smaller *k* value in the first non-significant Wilcoxon test was considered to be the best estimate of the number of subpopulations. Structure matrix (*Q* matrix) with one column less than the number of subpopulations was used in mixed model to achieve linear independence (STICH *et al.* 2008). ZHAO *et al.* (2007) applied a linear mixed model analysis by using Principal Component Analysis (PCA) instead of *Q* matrix and found it more effective model as it is not computationally intensive as the *Q* matrix method. We used PROC PRINCOMP in SAS 9.2® for our PCA. Principal components (PC) that explain 50% of the variation (*P* matrix) were used in the mixed model instead of the population structure matrix.

Kinship matrix (K) calculated in SPAGeDi (HARDY and VEKEMANS 2002) was used to calculate kinship coefficients as described by LOISELLE *et al*. (1995). Negative kinship values between inbreds were set to zero as described in YU *et al.* (2006). Based on suggestion of BERNARDO (1993), we calculated the kinship coefficient K_{ij} between inbreds i and j (*i.e.*, the probability that inbreds i and j carry alleles at the same locus that are identical by descent) on the basis of marker data according to

Kij=S−1 1−Tij+1

where S_{ij} is the proportion of marker loci with shared variants between inbreds i and j and T_{ij} is the average probability that a variant from one parent of inbred i and a variant from one parent of inbred j are alike in state, given that they are not identical by descent. Thus, T_{ij} is a function of the proportion of variants common to unrelated inbreds and is specific for each pair of inbreds (LYNCH 1988). Since the value of T_{ij} is unknown, T = 0, 0.05, ..., 0.95 (20 steps) were examined to obtain a REML estimate of T. Negative kinship values between inbreds were set to 0 as described in YU *et al.* (2006).

For the Pedigree (G) model we calculated the kinship matrix for the inbreds using the information from pedigree records, as described by FALCONER and MACKAY (1996), using PROC INBREED in SAS 9.2. The co-ancestry between unrelated individuals was set to zero (BERNARDO *et al.* 1996). The statistical analysis for the mixed model was performed in SAS 9.2 ® using PROC MIXED.

RESULTS:

The association mapping was conducted on the entire data set as well as the lines derived from crosses of the same Tunisian resistant source by dividing the data to different panels. For the entire data set the number of subpopulations explained by STRUCTURE was four (*k*=4). However number of subpopulations calculated for Tun34 and Tun7 sub-data set was five (*k*=5). For the entire data set the first two principal components explain only 14.5 and 7.4% of variability. Therefore, a total of 13 PCs that explained 50% of variability were used for mixed model analysis. For the Tun34 and Tun7 sub-data set these numbers reduced to 9 and 8 PCs, respectively.
The Kinship matrix (K) was calculated by SPAGeDi (HARDY and VEKEMANS 2002) as described in the methods. For the entire panel, MSD between the observed and expected *P*-values were plotted in K_T, QK_T, and PK_T models (FIGURE 1A). Association mapping methods that adhere to the nominal α-level show a uniform distribution of *P*-values. Therefore

the high MSD between observed and expected *P*-values of all marker loci shows the strong deviation of the observed *P*-values from the uniform distribution, which indicates that the empirical Type I error rate of these approaches is considerably higher than the nominal α-level (STICH *et al.* 2008). For this panel the MSD between observed and expected *P*-values for the K model ranged from 0.0155 to 0.0003, which was minimum with T-value equal to 0.65. However, the minimum MSD value for QK and PK models was minimum when the T was 0.70 and 0.65, respectively. For the Tun34 panel the MSD between observed and expected *P*-values was minimum at T-value of 0.55 for the K model. However, it was minimum at T-value of 0.30 for QK and PK models. For Tun7 panel MSD was minimum at T-value of 0.9 for K model while it was minimum at T-value of 0.65 for QK model and T-value of 0.55 for PK model. For the entire data the MSD value of the $K_{T(0.65)}$ and $QK_{T(0.65)}$ was the lowest. These two models have a MSD value at least 50 times lower than the Naïve model (FIGURE 1A). From the p-p plot (FIGURE 1B), it can be inferred that the mixed model performs better than the Naïve model.

References:

BERNARDO, R., 1993 Estimation of coefficient of coancestry using molecular markers in maize. Theor. Appl. Genet. **85:** 1055-1062.

BERNARDO, R., A. MURIGNEUX and Z. KARAMAN, 1996 Marker-based estimates of identity by descent and alikeness in state among maize inbreds. Theor. Appl. Genet. **93:** 262-267.

FALCONER D. S., and T. F. C. MACKAY, 1996 Introduction to quantitative genetics. Longmans Green, Harlow, Essex, UK.

HARDY, O. J., and X. VEKEMANS, 2002 spagedi: a versatile computer program to analyse spatial genetic structure at the individual or population levels. Molecular Ecology Notes **2:** 618-620.

LOISELLE, B. A., V. L. SORK, J. NASON and C. GRAHAM, 1995 Spatial Genetic Structure of a Tropical Understory Shrub, Psychotria officinalis (Rubiaceae). American Journal of Botany **82:** 1420-1425.

LYNCH, M., 1988 Estimation of relatedness by DNA fingerprinting. Mol. Biol. Evol. **5:** 584-599.

PRITCHARD, J. K., M. STEPHENS and P. DONNELLY, 2000 Inference of population structure using multilocus genotype data. Genetics **155:** 945-959.

STICH, B., J. MOHRING, H.-P. PIEPHO, M. HECKENBERGER, E. S. BUCKLER *et al.*, 2008 Comparison of mixed-model approaches for association mapping. Genetics **178:** 1745-1754.

WANG, J., P. E. MCCLEAN, R. LEE, R. J. GOOS and T. HELMS, 2008 Association mapping of iron deficiency chlorosis loci in soybean (Glycine max L. Merr.) advanced breeding lines. Theor. Appl. Genet. **116:** 777-787.

YU, J., G. PRESSOIR, W. H. BRIGGS, I. VROH BI, M. YAMASAKI *et al.*, 2006 A unified mixed-model method for association mapping that accounts for multiple levels of relatedness. Nat Genet **38:** 203-208.

ZHAO, K., M. J. ARANZANA, S. KIM, C. LISTER, C. SHINDO *et al.*, 2007 An *Arabidopsis* example of association mapping in structured samples. PLoS Genet **3:** e4.

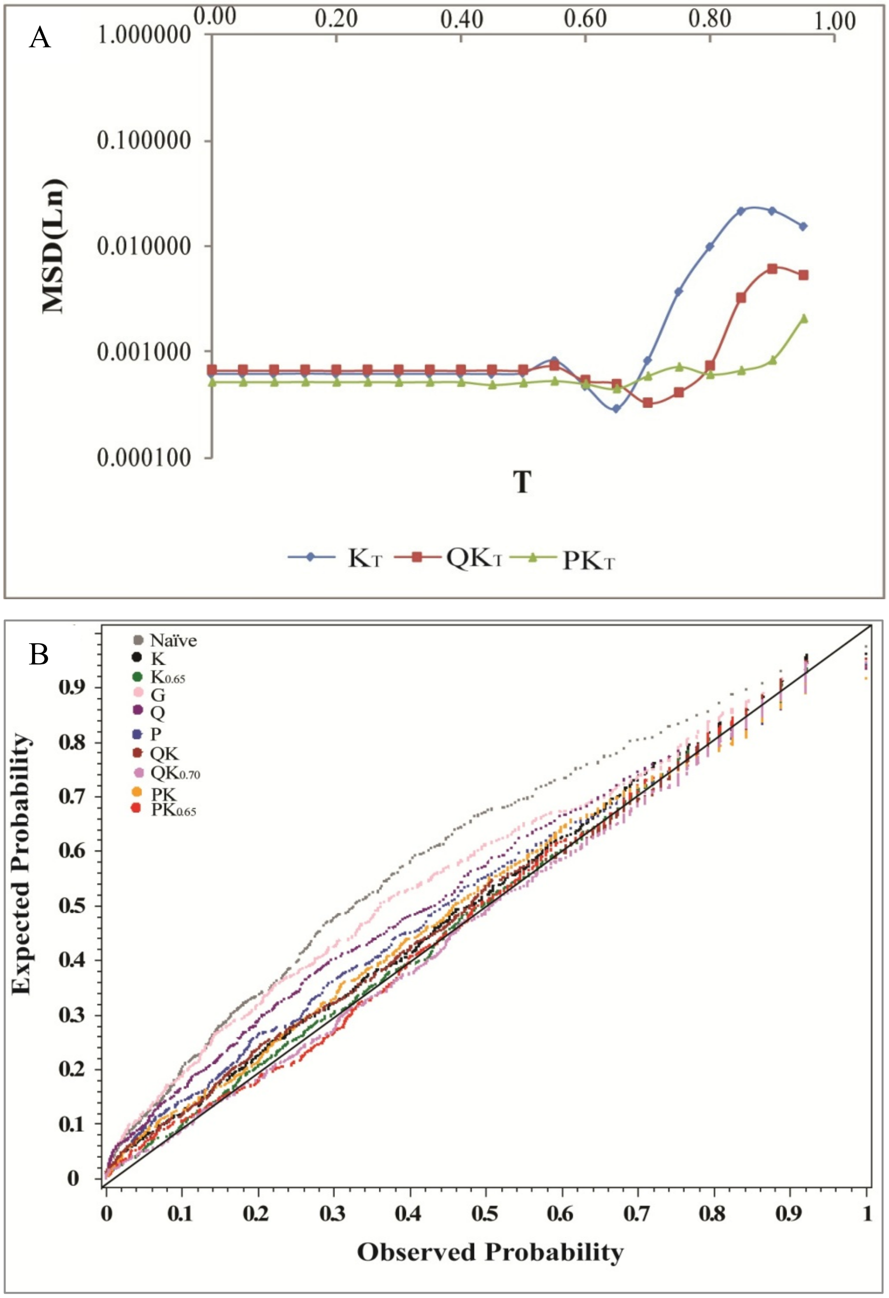

Figure 1 Mean of the squared differences (MSD) between observed and expected *P*-values for three different association mapping models for all the 20 measures for T-value (A). Simulation of Type I error produced under Naïve, simple and mixed models using 537 DArT markers (B).

Table 1 Mixed-model methods used for association mapping in this study and the statistical models describing them.

Model	Statistical model	Population Structure	Kinship
Naïve	$y = X\alpha + \varepsilon$		
K	$y = X\alpha + K\nu + \varepsilon$		SPAGeDI
K_T	$y = X\alpha + K\nu + \varepsilon$		T=0.65
G	$y = X\alpha + K\nu + \varepsilon$		Pedigree
Q	$y = X\alpha + Q\beta + \varepsilon$	STRUCTURE	
P	$y = X\alpha + P\beta + \varepsilon$	13 PC (explain 50% var)	
QK	$y = X\alpha + Q\beta + I\nu + \varepsilon$	STRUCTURE	SPAGeDI
QK_T	$y = X\alpha + Q\beta + I\nu + \varepsilon$	STRUCTURE	T=0.70
PK	$y = X\alpha + P\beta + I\nu + \varepsilon$	13 PC (explain 50% var)	SPAGeDI
PK_T	$y = X\alpha + P\beta + I\nu + \varepsilon$	13 PC (explain 50% var)	T=0.65

y is a vector of phenotypic values, β is a vector of fixed effects regarding population structure, α is the fixed effect for the candidate marker, *v* is a vector of the random effects pertaining to co-ancestry, and ε is a vector of residuals. *P* is a matrix of the significant principal component vectors that explain 50% variability. Q is a structure matrix with one column less than the number of subpopulations to achieve linear independence. *X* is the vector of genotypes at the candidate marker, and *I* is an identity matrix. The variances of the random effects are assumed to be $Var(v) = 2KVg$ and $Var(e) = IVR$, where *K* is the kinship matrix , *V*g the genetic variance, and *V*R the residual variance.

File S2
Supporting Data

File S2 is available for download as an Excel file at
http://www.g3journal.org/lookup/suppl/doi:10.1534/g3.111.000489/-/DC1/FileS2.xlsx

Table S1 Analysis of variance of infection rate for Type II FHB resistance measured in greenhouse in two seasons in 2006 and 2007.

Source	df	MS	*F* value	Pr>*F*
Season	1	0.12715	6.80	0.0110
Rep (Season)	7	0.01076	0.58	0.7736
Cultivar	11	0.09433	5.04	0.0001
Season × Cultivar	11	0.01530	0.82	0.6219
Error	76	0.01869		

Table S2 Mean of the squared differences (MSD) between observed and expected *P*-values for various association mapping models. The models tested in three different panels and the best model in each panel was picked based on the least MSD values and percentage of observations (obs) under 5% (Bolded).

Model	Entire data set		Tunisian 34 panel		Tunisian 7 panel	
	MSD	% of obs < 0.05	MSD	% of obs < 0.05	MSD	% of obs < 0.05
Naïve	0.01547	0.11732	0.03219	0.25000	0.001560	0.034211
K	0.00060	0.07632	0.00201	0.13119	0.001636	0.043614
K_T	**0.00029**	**0.05400**	0.00045	0.08416	0.000395	0.050000
G	0.00815	0.12570	0.00632	0.10670	NC	NC
Q	0.04444	0.09870	0.01026	0.15594	0.001258	0.105263
P	0.00182	0.09125	0.00268	0.09653	0.001601	0.047368
QK	0.00056	0.07635	0.00482	0.11911	0.001082	0.054496
QK_T	**0.00033**	**0.05028**	0.00039	0.07426	0.000955	0.092348
PK	0.00140	0.08380	0.00216	0.08376	0.001096	0.048913
PK_T	0.00045	0.06518	**0.00032**	**0.05941**	**0.000362**	**0.025424**
