# Supplementary material for: Mixed Model Association Mapping for Fusarium Head Blight Resistance in Tunisian-Derived Durum Wheat Populations
Source: G3 (Bethesda). 2011 Aug 1;1(3):209–18. doi: 10.1534/g3.111.000489 (PMC3276138; doi:10.1534/g3.111.000489)
Supplement: Supporting Information [file supp_1.3.209_FigureS1.pdf]

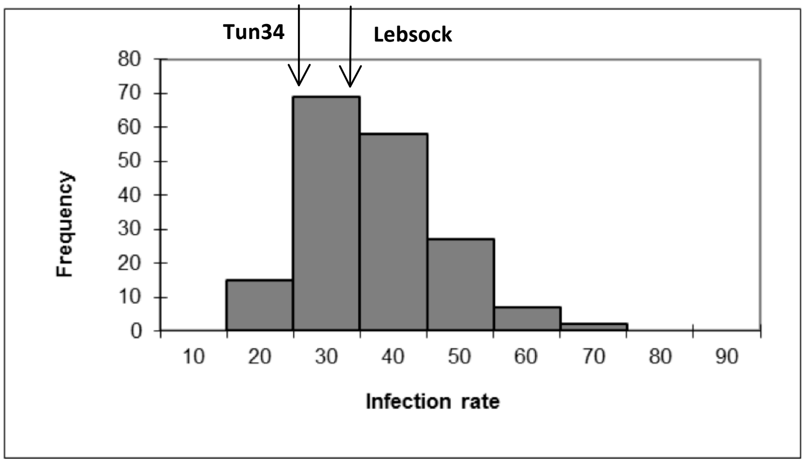

Figure S1 Frequency distribution of FHB severity among 169 BC_1F_6 wheat RILs of the Tun 34×Lebsock cross measured in the two greenhouse seasons in 2006 and 2007.
