# Supplementary material for: Mixed Model Association Mapping for Fusarium Head Blight Resistance in Tunisian-Derived Durum Wheat Populations
Source: G3 (Bethesda). 2011 Aug 1;1(3):209–18. doi: 10.1534/g3.111.000489 (PMC3276138; doi:10.1534/g3.111.000489)
Supplement: Supporting Information [file supp_1.3.209_TableS1.pdf]

Table S1 Analysis of variance of infection rate for Type II FHB resistance measured in greenhouse in two seasons in 2006 and 2007.

Source	df	MS	*F* value	Pr>*F*
Season	1	0.12715	6.80	0.0110
Rep (Season)	7	0.01076	0.58	0.7736
Cultivar	11	0.09433	5.04	0.0001
Season × Cultivar	11	0.01530	0.82	0.6219
Error	76	0.01869		
