# Supplementary material for: Mixed Model Association Mapping for Fusarium Head Blight Resistance in Tunisian-Derived Durum Wheat Populations
Source: G3 (Bethesda). 2011 Aug 1;1(3):209–18. doi: 10.1534/g3.111.000489 (PMC3276138; doi:10.1534/g3.111.000489)
Supplement: Supporting Information [file supp_1.3.209_TableS2.pdf]

Table S2 Mean of the squared differences (MSD) between observed and expected *P*-values for various association mapping models. The models tested in three different panels and the best model in each panel was picked based on the least MSD values and percentage of observations (obs) under 5% (Bolded).

Model	Entire data set		Tunisian 34 panel		Tunisian 7 panel	
	MSD	% of obs < 0.05	MSD	% of obs < 0.05	MSD	% of obs < 0.05
Naïve	0.01547	0.11732	0.03219	0.25000	0.001560	0.034211
K	0.00060	0.07632	0.00201	0.13119	0.001636	0.043614
K_T	**0.00029**	**0.05400**	0.00045	0.08416	0.000395	0.050000
G	0.00815	0.12570	0.00632	0.10670	NC	NC
Q	0.04444	0.09870	0.01026	0.15594	0.001258	0.105263
P	0.00182	0.09125	0.00268	0.09653	0.001601	0.047368
QK	0.00056	0.07635	0.00482	0.11911	0.001082	0.054496
QK_T	**0.00033**	**0.05028**	0.00039	0.07426	0.000955	0.092348
PK	0.00140	0.08380	0.00216	0.08376	0.001096	0.048913
PK_T	0.00045	0.06518	**0.00032**	**0.05941**	**0.000362**	**0.025424**
